# Lifelong Impacts of Moderate Prenatal Alcohol Exposure on Neuroimmune Function

**DOI:** 10.3389/fimmu.2018.01107

**Published:** 2018-05-22

**Authors:** Shahani Noor, Erin D. Milligan

**Affiliations:** Department of Neurosciences, School of Medicine, University of New Mexico Health Sciences Center, Albuquerque, NM, United States

**Keywords:** glia, inflammation, cytokines, spinal cord, neuropathy

## Abstract

*In utero* alcohol exposure is emerging as a major risk factor for lifelong aberrant neuroimmune function. Fetal alcohol spectrum disorder encompasses a range of behavioral and physiological sequelae that may occur throughout life and includes cognitive developmental disabilities as well as disease susceptibility related to aberrant immune and neuroimmune actions. Emerging data from clinical studies and findings from animal models support that *very low to moderate levels* of fetal alcohol exposure may reprogram the developing central nervous system leading to altered neuroimmune and neuroglial signaling during adulthood. In this review, we will focus on the consequences of low to moderate prenatal alcohol exposure (PAE) on neuroimmune interactions during early life and at different stages of adulthood. Data discussed here will include recent studies suggesting that while abnormal immune function is generally minimal under basal conditions, following pathogenic stimuli or trauma, significant alterations in the neuroimmune axis occur. Evidence from published reports will be discussed with a focus on observations that PAE may bias later-life peripheral immune responses toward a proinflammatory phenotype. The propensity for proinflammatory responses to challenges in adulthood may ultimately shape neuron–glial-immune processes suspected to underlie various neuropathological outcomes including chronic pain and cognitive impairment.

## Introduction

Exposure to alcohol during gestation can lead to a constellation of mild to severe disabilities that include cognitive (i.e., intellectual ability, learning, and memory) and behavioral (e.g., mood, attention, and impulse control) sequelae representing a continuum referred to as fetal alcohol spectrum disorder (FASD). Despite nearly 40 years of clinical studies and research in animal models demonstrating *in utero* alcohol exposure acts as a teratogen with very broad and long-term adverse effects, a recent study estimates that globally, about 10% of women in the general population consume alcohol during pregnancy. In discrete regions/countries, the percentage of women who consume alcohol while pregnant is much more (~46%) (1, 2).

While the most profound and most widely known consequences of prenatal alcohol exposure (PAE) encompass clearly identifiable neurobehavioral outcomes, more recent reports are uncovering PAE’s far more subtle and insidious lifelong effects on neuroimmune function. Studies examining altered neuroimmune responses as a consequence of PAE are shedding light on potential underlying molecular mechanisms associated with PAE-induced neurological dysfunction (3–6). In studies utilizing animal models of PAE, robust neuroimmune activation such as heightened proinflammatory cytokine production is observed in the neonatal and adult brain (3, 4, 6–12). Often, these animal models include high and/or chronic prenatal and neonatal alcohol exposure to mimic the effects of binge/heavy drinking during and after (during lactation) pregnancy observed in humans. Additionally, albeit more sparse, clinical and controlled animal studies have been conducted to address whether comparatively less frequent or moderate gestational alcohol exposure exerts similar effects on neuroimmune function. In this review, the focus is predominantly on studies modeling moderate or low PAE and the effects of this exposure on the neuroimmune axis. In this context, discussion points will highlight the possible role PAE may play in shaping the inflammatory response in the central nervous system (CNS).

## FASD Spans a Range of Deficits from Severe to Mild

### Early Clinical Observations of Fetal Alcohol Syndrome (FAS) and the Evolution of FASD

Birth anomalies resulting from PAE were first described as FAS in 1973 by Jones and colleagues (13, 14). Since these seminal reports, the FAS criteria and diagnostic schemas have been refined. FAS is considered the most serious consequence of high levels of PAE and is distinguished from less overt outcomes that are encompassed by FASD. FAS can include significant pre- and postnatal growth delays and a characteristic pattern of craniofacial abnormalities. Additionally, FAS-associated defects have been observed in a variety of organ systems such as the visual, auditory, cardiac, and urogenital systems (15). However, since the first description of FAS, it has become profoundly clear that not all individuals exposed to high levels of prenatal alcohol reveal overt dysmorphia. Other less clearly defined neuropathological conditions inclusive of cognitive and behavioral deficits are now recognized outcomes of FAS. The range and magnitude of cognitive and behavioral deficits vary, which are likely influenced by many factors including alcohol exposure experienced during discrete developmental periods, mother’s alcohol consumption pattern, the nutrition status of the mother, as well as genetic factors (16–19). Therefore, the umbrella term FASD has been developed to better capture the complexity (e.g., magnitude and pattern of alcohol consumption) and wide-ranging consequences of PAE that includes the more severe FAS. Notably, FASD encompasses various diagnostic conditions that not only capture FAS, but also partial FAS, alcohol-related neurodevelopmental disorder, and alcohol-related birth defects (20, 21). The neurological sequelae present in individuals across the spectrum of FASD now incorporates cognitive impairments such as deficits in learning and memory, executive and motor function, attention and behavioral problems inclusive of psychiatric and substance abuse disorders, and diminished skills related to social interaction (22). Moreover, a number of studies suggest that the effects of FASD may alter bodily systems such as the immune system that is known to impact neurological function.

Although immune dysfunction is not considered diagnostic of FASD, multiple clinical reports and case studies indicate that children with FASD frequently face secondary medical disabilities related to immune dysregulation (i.e., autoimmune or inflammatory reactivity). For example, FAS children have high rates of upper respiratory infection and recurrent serious otitis media (middle ear inflammation). Additionally, children diagnosed with FAS and neonates prenatally exposed to alcohol experience a high incidence of infection and immune-related pathologies, such as urinary tract infection, meningitis and the chronic autoimmune neuromuscular disease, myasthenia gravis (23). Furthermore, maternal alcohol consumption increases circulating proinflammatory cytokine exposure to the fetus (24). Because alcohol levels persist longer in the blood of the fetus than in the mothers’ (25), it is possible that circulating fetal proinflammatory cytokines from alcohol exposure reprogram inflammatory responses long after birth. PAE with consequent increased fetal proinflammatory cytokine levels may underlie some of the cognitive impairments seen with FASD, as discussed further below. However, human studies assessing CNS-specific neuroimmune parameters in FASD individuals are rare due to limited methodologies and access to samples. The majority of human clinical studies of adverse neurological outcomes and aberrant immune competence are from self-reports of heavy drinking mothers or mothers with children diagnosed with FAS.

### Human Moderate PAE Studies

A surprisingly limited number of human studies address the causal effect of moderate drinking (i.e., 1 standard drink or 14 g of ethanol per day) during pregnancy and later outcomes of these children (26). Of the few clinical studies aimed to address this important public heath question, a recent prospective cohort study was conducted that included women who consumed light to moderate alcohol levels that did not report binge drinking. The behavioral trajectories of the children were followed. This study reported that the children of mothers’ who reported moderate drinking during pregnancy experienced an increased risk for early childhood behavioral problems (e.g., getting along with other children in a group) (27). A comprehensive systemic review by Mamluk et al. concluded that due to the paucity of well-designed human studies, it remains unclear whether a safe limit of alcohol consumption during pregnancy exists (28). More recently, Muggli et al. suggested that even low levels of PAE can influence craniofacial development, thus supporting that abstinence from alcohol while pregnant is the safest option (29). In an attempt to address the gap in knowledge of the clinical CNS developmental consequences from low/moderate PAE, a number of well-controlled studies applying animal models have been reported. Indeed, the application of low to moderate PAE in animal experiments has significantly advanced our understanding of the potential direct and indirect effects of PAE on the CNS inflammatory response.

## Animal Models of PAE Reveal Basal Neuroimmune Changes

A number of published reports have modeled the impact of moderate PAE on the developing and adult CNS in animals (12, 26, 30–32). Studies in rodent models demonstrate long-lasting neurobehavioral deficits caused by moderate levels of alcohol exposure during prenatal (first and second trimester pregnancy) and neonatal (equivalent to the third trimester of human pregnancy) development. Abnormalities in learning, memory, motor coordination, social behavior, and stress responses were observed. Notably, these behavioral alterations are associated with impairments in neurotransmitter systems, neuromodulators, and synaptic plasticity in several brain regions (26). In studies aiming to generate moderate PAE, pregnant dams achieve blood alcohol levels within the range of 0.08–0.17 g/dl (80–170 mg/dl). For reference, 0.08 g/dl is considered the US legal intoxication limit. One aspect of these earlier studies that came to light is the effect of moderate PAE on CNS-immune interactions.

While collective evidence from many animal studies suggest that moderate or low-alcohol exposure can persistently alter multiple neurotransmitter and neuromodulatory systems (26), few studies have demonstrated that moderate PAE affects neuroimmune function. Data have been ambiguous regarding the CNS outcomes from developmental low-dose alcohol exposure with several previous studies suggesting moderate PAE might be less disruptive to neurodevelopment and the neuroimmune system than binge-like exposures (33–35). However, a lack of comparative studies exist that investigate neuroimmune parameters and neurodevelopment following discrete trimester and pattern of alcohol exposure (binge versus moderate). Despite the brevity of needed basic science studies, the following section will discuss evidence to date that point to possible mechanisms underlying the impact that PAE exerts on neuroimmune function during CNS development, the early postnatal period and in adulthood.

Immune reactions within the CNS are initially and predominantly mediated by glial cells, often with engagement of infiltrating innate/adaptive immune cells from the periphery (36). While glial cells (microglia and astrocytes) play key roles in neuronal homeostasis in the CNS, they are also primary immune responders in the CNS (37, 38). Upon immune activation, glial cells in the adult brain secret proinflammatory cytokines such as interleukin (IL)-1β, IL-6, tumor necrosis factor (TNF), cyclooxygenase 2, and nitric oxide as well as anti-inflammatory factors such as IL-10 (39–41). Neuroinflammatory agents from glial cells stimulate neurons and infiltrating immune cells that in turn secrete chemokines including monocyte chemotactic protein-1 [MCP-1, also known as C–C motif chemokine ligand-2 (CCL2)], thereby furthering neuroinflammatory processes that contribute to neuropathologies associated with many degenerative and neuroinflammatory disorders (42–44).

While the animal model for moderate PAE is the primary focus of this review, most of our knowledge of neuroimmune responses following alcohol exposure has come from experiments that model binge-drinking where sporadic and high levels of developmental alcohol exposure occurs. In consideration of these animal models, the data reveal that alcohol exposure during early postnatal development in rodents (a third trimester equivalent in humans) results in microglial and neuronal loss, microglial, and astrocyte activation (7, 45) and increased proinflammatory cytokine and chemokine expression in diverse brain regions such as the hippocampus, cerebellum, and cerebral cortex (4). Moreover, neuroinflammation in the fetus has been verified (46, 47). These results suggest a link exists between glial function and ethanol-induced neuropathology. Indeed, other studies reveal that the adverse effects of alcohol on CNS glial activation are mitigated by an anti-inflammatory agent, a peroxisome proliferator-activated receptor gamma agonist (4, 7) that blocks microglial activation.

A recent report by Pascual et al. examined the neuroimmune effects of *moderate* prenatal and postnatal alcohol exposure. In this model, pre-pregnant female breeder mice received 10% ethanol (v/v) in their drinking water for 2 months before mating resulting in an average peak blood alcohol concentration (BAC) of 125 ± 20 mg/dl. After 2 months, these pre-pregnant females were placed with male breeders, and following pregnancy, dams continued to receive 10% ethanol solution throughout gestation and lactation, with weaning occurring at postnatal day (PND) 25 (48). Tissues were collected from discrete subgroups representing specific timepoints. Cerebral cortices were collected at embryonic day 15 (E15), as well as on PND 0, 20, and 66 followed by an examination of key proinflammatory cytokines and chemokines. This study demonstrated increased IL-1β, IL-17, macrophage inflammatory protein (MIP)-1α, and fractalkine protein levels in fetal cerebral cortex at E15 (48). In this study, sex differences were not determined at this timepoint. These cytokines remained elevated at birth (PND 0), along with increased CCL2 (MCP-1), but returned to normal levels by PND 20 in females; however, the pattern observed in males is distinctly different, as discussed below. Additionally, IL-1β protein levels remained consistently and significantly elevated through PND 66, as cortex was examined at E15, PND 0, 20, and 66. Increased protein levels of CD11b (integrin alpha M chain) and major histocompatibility complex 2 (MHC2) (at PND 0 and 20) were also detected in the brain (48), suggesting an increase in myeloid cell activation/activity.

However, as alluded to above, interesting sex differences were observed in these neuroinflammatory effects, with increased levels of MCP-1 and MIP-1α only detected at PND 20 in male pups without an increase at PND 0, suggesting the basal postnatal expression of these chemokines is influenced by as yet unrecognized postnatal gender-specific factors, with cytokine expression patterns programmed during prenatal development. In support of this possibility, increases in female-derived CCL2 and MIP-1α protein levels were observed significantly sooner (PND 0) but, plummeted by the third week of postnatal development (PND 20) compared with levels observed in males. Additional sex differences in cytokine levels, as no changes in IL-1β levels were measured in male pups with pre- and postnatal alcohol exposure. Overall, these sex differences were associated with increased neuroinflammation in female pups in this particular model of pre- and postnatal alcohol exposure (48).

In a third model of PAE, pregnant dams were administered alcohol (using a flexible gavage catheter, 2 g/kg of ethanol, twice daily) only during gestational days 10–16, with dam’s peak BACs reaching 70 mg/dl (12). This study showed robust increases in cytokines and chemokine/chemokine receptors such as CCL2, 3, and 6, C–C motif chemokine receptor 2 (CCR2) (the receptor for CCL2), CCR6, IL-21, IL-10, and TNF mRNA in the fetal hippocampus and cortex at E17. An additional important finding from this report demonstrated that the alcohol-induced effects on the maternal immune system appear to be minimal, while the placenta and the developing fetal brain mount rapid (~24 h) and robust immune responses as a consequence of moderate PAE (12). These results from PAE models are further supported by other reports where animal studies of perinatal cytokine and chemokine increases within the fetal brain co-occur with impaired neonatal brain white matter microstructural integrity as well as motor dysfunction in offspring (49, 50). Combined, these reports suggest that elevated circulating proinflammatory factors (elicited either by immune stimulation or by PAE) during pre- and perinatal periods may significantly contribute to brain neuropathology in young offspring.

Intriguingly, these seminal reports on the impact of PAE on neuroimmune function revealed distinctly different observations of neuroimmune responses between males and females. Specifically, Terasaki and Schwarz (12) reported that acute (~24 h) neuroinflammatory gene activation occurring in response to low levels of PAE during early fetal brain development are sex specific. For a number of immune molecules such as CCL2 and IL-5, the effects of PAE were dependent on the sex, because their expression levels were generally decreased in males but increased in females with PAE. Overall, female pups (E17) with PAE revealed higher levels of inflammatory gene expression in the brain compared with their male counterparts (12).

In addition to the PAE sex-specific differences in chemokines/cytokines, the same report demonstrated increased levels of CD11b and brain-derived neurotropic factor in adult brain examined on PND 90 (51). These data indicate that long-term immune activation in different brain regions does not require a parallel induction of cytokines. In a separate study utilizing moderate PAE, increases in adolescent hypothalamic CCR2 receptor was reported (10). Observations from our laboratory demonstrate that low-dose alcohol exposure throughout gestation in rats (peak dam BAC ~60 mg/dl) resulted in changes in immune cell markers in adult offspring. For example, increased expression of β-integrin adhesion molecules (e.g., CD11a and CD29) on CD11b^+^ cells collected from spinal cord was measured. Increased expression of these adhesion molecules may reflect altered/impaired regulation and/or hypersensitivity to immune stimuli by immune cells and/or microglia (52).

As noted above, greater levels of alcohol exposure during early postnatal development in rodents (a third trimester equivalent in humans) results in microglial and astrocyte activation and microglial loss (7, 45). However, microglial *loss* has not been reported following *moderate* PAE. Rather than microglial loss, published data show moderate PAE induces early neuroinflammatory responses in the fetal brain, and while speculative, early developmental CNS inflammation may affect the pre- and postnatal roles of microglia on neuronal development. Moreover, PAE-induced activation of microglia may cause persistent changes in their activation status and function resulting in neuroimmune and neurobehavioral consequences following a subsequent challenge (immune or tissue damage) in adulthood. In support of this possibility, a growing body of evidence, as discussed in the following sections highlights the potential long-term impact of moderate PAE in shaping CNS responses to subsequent challenges.

## Moderate PAE Shapes Neuroimmune Responses to Subsequent Inflammatory Challenge Later in Life

### CNS Immune Responses Upon Brain Injury

While speculative, glial cells may remain primed following an initial stimulation during gestational development (i.e., PAE), with classic activation markers returning to basal levels. However, these previously stimulated glia may develop an increased propensity for enhanced responses following subsequent immune stimulation (52, 53). That is, it is possible under some circumstances that classic glial activation markers are uncoupled from their primed state such that basal levels of these markers are observed, yet these same glia over-respond to normal stimuli. For example, PAE primes the CNS glial-immune response as observed by enhanced inflammatory cytokine production following subsequent immune challenge during adulthood. The observation that fetal exposure to alcohol alters responses of glial and immune cell factors to CNS injury in adults was first described by DeVito and Stone (54). In this study, animals were exposed to moderate levels of ethanol (46–101 mg/dl) *in utero*. The PAE offspring were maintained until adulthood and underwent discrete cortical damage *via* a stab injury. Data show increased vascular cell adhesion molecule-1 (VCAM-1) and cluster of differentiation 68 (CD68) on both microglia and macrophages in PAE animals, indicating brain endothelial cell activation in addition to microglia and/or macrophage activation. Interestingly, glial fibrillary acidic protein (GFAP), a marker of astrocyte activation, and proinflammatory TNF production were diminished in PAE rats compared with the experimental controls in this brain region 4 days following injury. However, re-exposure to alcohol several days before and after the stab wound surgery further augmented CD68 and VCAM-1 expression in adult brains of PAE animals (54). These data suggest moderate PAE has the potential to alter and augment key components of neuroimmune responses to subsequent CNS injury in adulthood.

### Neuroimmune and Cognitive Outcomes in PAE Adults Following Challenge With LPS

The hippocampus and cortex are critical brain regions for learning and recognition memory. These brain regions are also vulnerable to glial and immune activation (55). In the study by Terasaki et al., moderate PAE rats were given a systemic challenge on PND 90 with a low dose of lipopolysaccharide (LPS; 25 µg/kg, intraperitoneal injection) to induce an acute immune response. Notably, LPS expresses pathogen-associated molecular patterns signaling to immune cells that pathogen invasion has occurred. Within 4 h of LPS injection, exaggerated IL-1β and IL-6 protein levels were measured in the hippocampus of PAE male rats. In the medial prefrontal cortex (mPFC), PAE and LPS immune challenge generated additive IL-1β increases in both males and females. Greater levels of CD11b protein were also detected in adult mPFC of PAE males and females, while no changes were detected in the hippocampus (12). These proinflammatory cytokines are thought to play significant roles in cognitive and psychiatric disorders (56–58). Thus, cognitive function in adult PAE rats with adult-onset immune activation (with LPS) was investigated. Several important outcomes were observed from this report. First, data revealed that basal PAE (without LPS challenge) reduces performance on recognition memory tasks [e.g., novel object recognition (NOR)] in adult males and females. Second, upon mild immune activation in adulthood, profound recognition memory deficits were observed in PAE offspring (both males and females) (12).

In a more recent study, PAE rats were further exposed to an acute binge-like dose of alcohol in adulthood (51). This study revealed that PAE exaggerated IL-6 production in the prefrontal cortex following alcohol exposure in adulthood, suggesting that adult alcohol exposure may act as a neuroinflammatory agent. Interestingly, IL-1β and CD11b levels in the cortex were decreased in response to acute adult binge-like alcohol exposure regardless of PAE. In this same study, adult cognitive function was assessed by performance on hippocampal-dependent (novel object location/NOL) and non-hippocampal-dependent (NOR) behavioral tasks. Adult cognitive function was disrupted in PAE offspring with or without adult alcohol exposure. Minor sex differences were additionally observed. Females (with PAE alone) and males with PAE in combination with an acute binge-dose of alcohol were deficient in learning the NOL task. In addition, male PAE animals and PAE plus an acute binge-like dose of alcohol in adulthood (in both males and females) negatively impacted performance the NOR task (51). Together, these data highlight that low/moderate levels of PAE can negatively impact cognitive function in later life, and these long-term consequences can be exacerbated by subsequent challenges (e.g., binge-like doses of alcohol exposure or immune activation).

## Spinal Glial-Immune Responses That are Altered by Moderate PAE: Increased Vulnerability to Chronic Neuropathic Pain

Multiple studies now suggest that even low levels of PAE may pathologically prime CNS glial cells and disrupt their supportive role in neuronal function, not only during development, but also throughout adulthood. PAE may generate CNS susceptibility to injury through the actions of aberrant immune responses that ultimately act to exacerbate challenges in the CNS rather than sequester damage and enhance healing. Therefore, we hypothesize that the risk and severity of chronic neurological disorders is enhanced as a consequence of PAE. In recent years, our laboratory has examined the development of adult-onset chronic neuropathic pain in an animal model of moderate PAE to better elucidate the spinal cellular and molecular neuroimmune adaptations PAE induces.

### PAE Potentiated Spinal Immune Responses and Chronic Pain

An intriguing behavioral manifestation in children diagnosed with developmental disabilities including FASD is abnormal sensory function such as tactile hypersensitivity (59–63). In support of clinical observations, a longitudinal study in rhesus monkeys revealed that heightened sensitivity to light touch was significantly greater in adult PAE monkeys compared with control-treated monkeys (30). Curiously, tactile hypersensitivity is also frequently observed in people with peripheral nerve damage and is referred to as allodynia. From the perspective of pain transmission, allodynia results from aberrant synaptic communication in the spinal cord where incoming sensory transmission is relayed to higher brain areas. Thus, allodynia following peripheral nerve damage is a CNS disorder. Therefore, one hypothesis that quickly developed was whether the underlying cause for tactile hypersensitivity (allodynia) observed in individuals with FASD could be due to neurological dysregulation of spinal pain relays. Furthermore, many reports utilizing rodent models of peripheral nerve damage demonstrate that allodynia occurs as a consequence of over-active spinal astrocyte and microglial responses (39).

**Figure 1:**
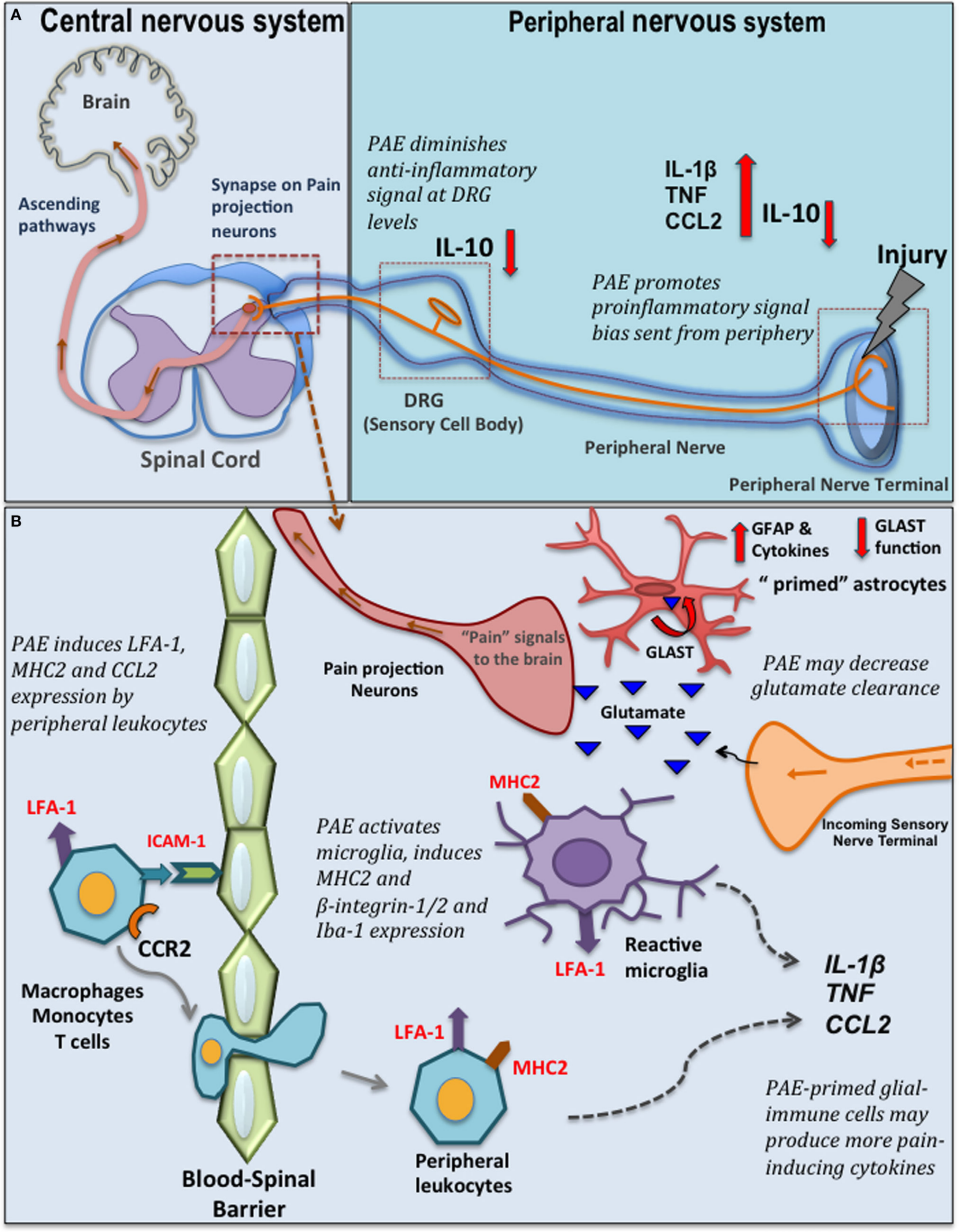
Prenatal alcohol exposure (PAE)-induced changes in spinal glial-immune interactions during chronic pain. **(A)** Peripheral nerve injury activates resident macrophages and Schwann cells that send out the danger signals, such as nitric oxide and chemokines to recruit peripheral immune cells (e.g., macrophages, mast cells, neutrophils, and T cells) that invade the lesioned site. These immune cells produce further proinflammatory cytokines/chemokines such as CCL2, tumor necrosis factor (TNF), interleukin (IL)-1β. Eventually, a compensatory increase of IL-10 is observed that dampens the peripheral inflammatory reactions at the lesion site. In addition, significant pain modulation and spinal glial-immune activity occurs at the level of the dorsal root ganglia (DRG) (where sensory neuron cell bodies are located) and spinal cord. Following nerve injury, satellite glial cells and infiltrating peripheral immune cells in the DRGs produce cytokines and further modulate sensory neuron (nociceptors relay painful noxious stimuli) activity and gene expression. Simultaneous significant microglia and astrocyte activation is observed in the dorsal horn where the primary afferent fiber terminals relay pain information to secondary pain projection neurons within the dorsal horn of the spinal cord. Neurotropic factors (brain-derived neurotropic factor) and cytokines produced by glia (satellite glia, microglia, and astrocytes) and peripheral immune cells at these discrete regions significantly modulate pain processing. PAE alters the inflammatory milieu at the peripheral lesion site and at the DRG promoting a bias toward proinflammatory signaling, with a simultaneous significant deficiency in IL-10 production that may contribute to the exaggeration of danger signals relayed from periphery to the central nervous system-immune system. **(B)** PAE augments production of CCL2 by peripheral leukocytes. CCL2–CCR2 mediated interactions may further activates LFA-1 to promote LFA-1-ICAM-1 mediated trans-endothelial leukocyte migration across the blood–spinal barrier. In the spinal cord, these peripheral leukocytes also produce various cytokines and interact with neurons and glial cells to further activate glia. Moreover, PAE is thought to prime glial cells, as evidenced by increased adhesion molecules and major histocompatibility complex 2 (MHC2) expression (52). Therefore, with nerve injury, PAE potentiates microglial and astrocytic activity, which may in turn lead to increased proinflammatory cytokine production in the spinal cord dorsal horn. Also, PAE may alter homeostasis of neurotransmitters (such as glutamate) by decreasing glutamate transporter expression, therefore activating pain projection nerve terminal *via* excessive glutamate. GLAST, glutamate aspartate transporter; CCR2, C–C motif chemokine receptor 2; CCL2, C–C motif chemokine 2; ICAM-1, intracellular adhesion molecule-1; LFA-1, leukocyte function-associated antigen-1; Iba-1, ionized calcium binding adaptor molecule; GFAP, glial fibrillary acidic protein.

As noted, a large body of evidence demonstrates that the neuro-glial-immune interface may underlie aberrant adult CNS function as a consequence of PAE. This background pointed to the hypothesis that PAE may exacerbate allodynia through elevated spinal glial actions. Therefore, a well-characterized rat model of low/moderate PAE (64) was utilized to determine whether enhanced allodynia and immune and glial responses occurred following peripheral nerve challenge in adult PAE offspring (52). The data indeed suggest several overlapping neuroimmune interactions are present between chronic pain and PAE. Figure 1 summarizes the proposed spinal mechanisms by which PAE potentiates chronic neuropathic pain based on current published reports.

A brief overview of chronic pathological pain will provide the appropriate context supporting the rationale for studies that explored spinal glial mechanisms underlying enhanced pain in PAE offspring. Chronic pathological pain often results from peripheral nerve damage, infection, or a combination of both trauma and inflammation. While chronic pathological pain involves hyperexcitability (sensitization) of pain projection neurons in the spinal cord or brainstem, critical roles of glial cells in the spinal cord, and satellite glial cells of the dorsal root ganglia (DRG) have been established (65, 66). Following injury of peripheral axons (e.g., sciatic nerve), excitatory pain transmitters are released by the nerve terminals projecting to the spinal cord that, in turn, synapse onto pain projection neurons. As noted above, the surrounding glial cells (Figure 1A) respond to these classic pain transmitters. The glial response includes release of IL-1β and TNF, and the chemotactic cytokine, CCL2/MCP-1 among a number of proinflammatory factors. Increased CCL2–CCR2 actions upon neuronal and glial activation in the spinal cord and DRG following peripheral nerve damage can result in leukocyte accumulation in discrete anatomical regions along the pain pathway (e.g., peripheral nerve axons, DRG, corresponding spinal cord regions, Figure 1B) (67–71). Over time, due to the feed forward actions of IL-1β and TNF and other cytokines released from activated glia and infiltrating immune cells, pathological sensitivity to non-painful stimuli develops (e.g., mechanical allodynia) resulting in chronic neuropathic pain (67, 72).

The crucial contributions of both protective and detrimental roles of active glial cells during conditions that lead to chronic painful neuropathy (39) necessitated further exploration of PAE-induced altered glial responses to peripheral nerve injury. Our recent work elucidates the impact of moderate PAE on spinal neuron–glia-immune interactions using an adult-onset peripheral nerve damage model of neuropathic pain (52). Chronic constriction injury (CCI) of the sciatic nerve is a widely used rodent model of sciatic trauma leading to neuropathy in which 4 snugly tied, but not pinching, chromic gut sutures are applied (73). In this study, CCI was applied to 4–5-month-old PAE male rats (equivalent to young adulthood). The data reveal that PAE potentiates allodynia. Moreover, this work demonstrates that even with moderate PAE, spinal microglia are primed, as indicated by increased CD11b expression. It was also observed that PAE, regardless of peripheral nerve damage, enriched the β2 integrin^+^ (leukocyte function-associated antigen) and β1-intergin^+^ and MHC2^+^ population of microglia and leukocytes in the lumbar spinal cord (52). Expression of these β-integrin adhesion molecules and MHC2 suggests involvement of microglial *and* immune cell activation, migration, antigen presentation, and primed functional responses (74, 75). While these data are indicative of PAE-induced priming of spinal glial and immune cells, further studies are needed to determine whether PAE-induced priming leads to increased activation of β-integrin on peripheral leukocytes (76) that is driven by increased CCL2-mediated interactions in PAE spinal cord.

Additional evidence exists supporting the possibility that PAE augments astrocyte and microglial activation in the dorsal horn of the spinal cord in neuropathic adult male rats. That is augmented expression of Iba-1, a well-characterized microglial activation marker, and GFAP (Figure 1B) were observed in PAE rats with CCI (52, 53). In line with these observations, analysis of cytokine levels in sham-treated PAE rats revealed that DRG IL-10 protein levels are remarkably suppressed, with IL-10 suppression greatest in PAE neuropathic rats compared with control-treated rats (52). It is important to note that spinal IL-10 is established to suppress allodynia in rodent models of peripheral neuropathy by blocking the spinal actions of a number of proinflammatory factors (77–80). In addition, prior reports demonstrate that DRG IL-10 protein levels are significantly reduced under neuropathic conditions, with satellite glia being a cellular source of DRG IL-10 (81, 82). Thus, in light of prior evidence, the current data suggest PAE further blunts the protective actions of basal DRG glial-derived IL-10 against proinflammatory actions (52). This work additionally suggests moderate PAE primes spinal microglia and astrocytes such that the response of these cells to subsequent damaged-self signals occurring during Wallerian degeneration from peripheral nerve damage is greatly enhanced. While pain relevant cytokine levels and their specific cellular sources (astrocytes, microglia, different subsets of T cells, and/or macrophages) in the spinal cord from neuropathic PAE rats is still under investigation, these data provide evidence that PAE generates stable, potentially lifelong spinal and peripheral nervous system glial, and immune cell hyper-reactivity following a second insult (e.g., localized sciatic nerve trauma) that is initiated in adulthood.

### Susceptibility to Developing Chronic Pain Following Minor Nerve Injury

One of the most compelling aspects of the data from offspring with enhanced neuropathy following moderate PAE is that their baseline sensory (light touch) responses are identical to non-PAE controls (52). This striking observation indicates that the insidious effects of PAE on glial–neuronal and immune responses are masked, and only following a second challenge (e.g., nerve injury or increased cytokine exposure), the adverse effects of PAE on neuroimmune responses are revealed. This observation suggests that neuropathological consequences of low/moderate PAE are not overt, but rather, PAE-related glial priming becomes evident only following a subsequent nerve injury or challenge (12, 52). That is, PAE may a be risk factor for developing chronic pathological CNS conditions in response to minor insults that typically go unnoticed in non-PAE individuals because they are resolved by a healthy neuroimmune response.

In support of the possibility that PAE is a risk factor for adult neuropathy, a recent investigation revealed that PAE renders one susceptible to developing pathological pain induced by a mild peripheral nerve injury, possibly through exaggerated immune and spinal astrocyte responses. PAE rats display potentiated allodynia following CCI (with four sutures) that was initiated in late adulthood (1-year-old rats). However, when CCI is reduced to a minor injury with only a *single* suture, robust allodynia was observed in PAE adults while touch sensitivity in control animals remained entirely unaltered (53). Surprisingly, neuropathic PAE animals from minor injury did not reveal significant spinal cord *microglial* activation by day 10 after CCI, as revealed by immunoreactivity for Iba-1 and transmembrane protein 119, both markers of microglial proliferation and/or activation, in the dorsal horn of the lumbar spinal cord (53, 83). Conversely, significant increases in *astrocyte* activation, as examined by GFAP immunoreactivity were observed in the spinal cord compared with non-neuropathic control rats. While, it is possible that increased microglial activation occurs earlier or later than the time points examined, these data suggest that spinal astrocytes and not microglia are playing a greater role in mediating the PAE-associated risks for developing allodynia following minor injury. Thus, PAE-induced pathological neuroimmune responses underlying abnormal CNS sensory processing can be unmasked by minor injury (53).

## Possible Mechanisms Underlying the Impact of PAE on the Neuroimmune Axis in Adults

**Figure 2:**
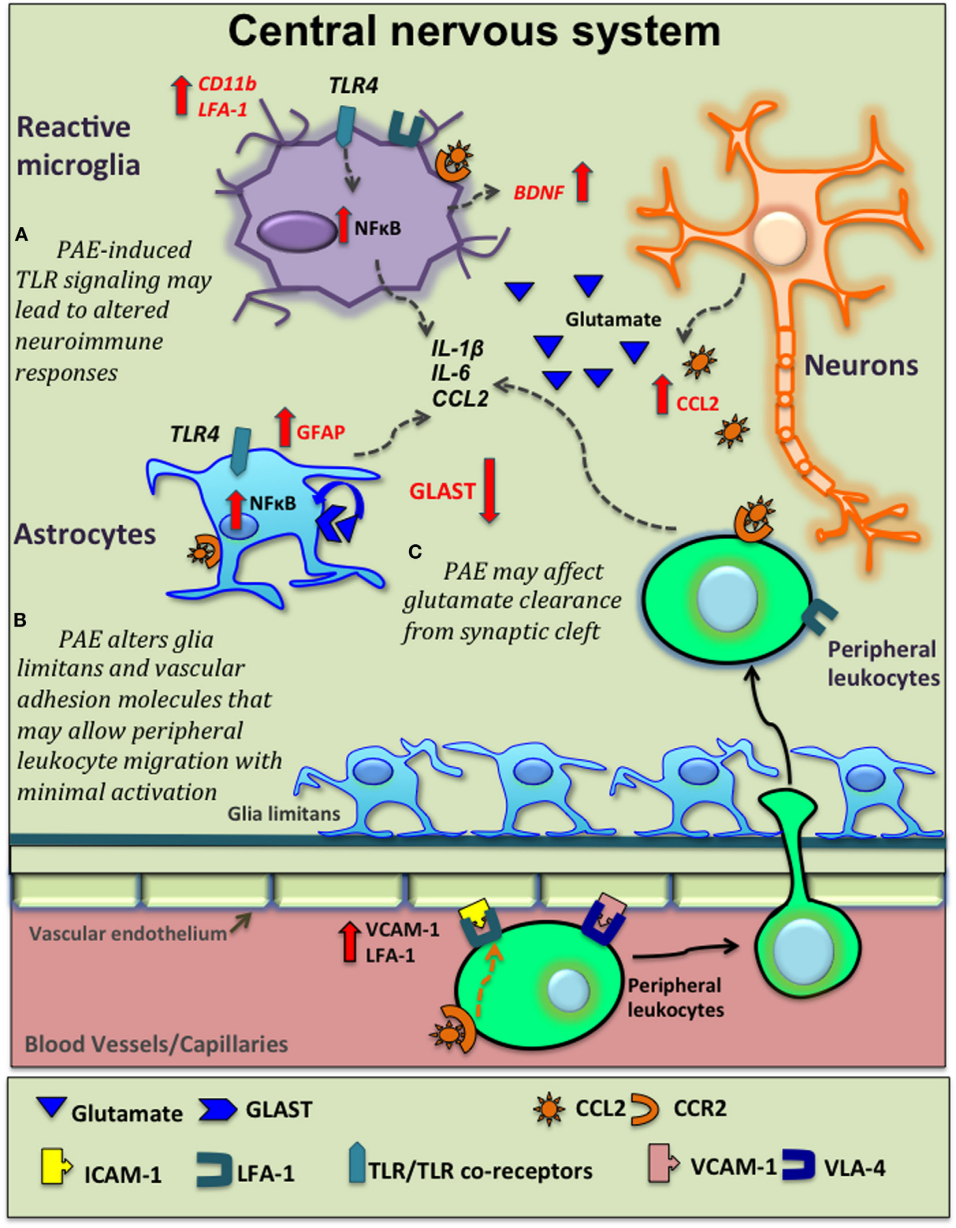
Potential venues of prenatal alcohol exposure (PAE) altering neuroinflammation. Moderate PAE leads to epigenetic alterations of neural gene expression that may extend to glial-immune cells altering their basal activation status and function (not shown here). Based on current supporting evidence, this schematic diagram shows several other potential mechanisms underlying PAE’s contribution to central nervous system (CNS) inflammation. **(A)** PAE-induced toll-like receptor 4 (TLR4)-mediated signaling activates the NFκB pathway in microglia and astrocytes leading to production of various inflammatory cytokine, as explored in high-alcohol exposure models. PAE-induced expression and function of TLRs may lead to altered neuroimmune responses followed by subsequent immune activation. **(B)** PAE increases C–C motif chemokine ligand-2 (CCL2) production in the CNS that may be released damaged neurons in the CNS and also *via* blood-brain-barrier (BBB). CCL2 acts on peripheral leukocytes to recruit them to the CNS. Glial cells also express C–C motif chemokine receptor 2 (CCR2) and produce inflammatory cytokines in response to CCL2. Similarly, PAE-induced increases in adhesions molecules such as vascular cell adhesion molecule-1 [VCAM-1, binds with very late antigen-4 (VLA-4) on leukocytes] and leukocyte function-associated antigen-1 (LFA-1), in conjunction with altered morphology of glia limitans, may facilitate and increase the magnitude of peripheral leukocyte migration across BBB following subsequent immune activation. **(C)** PAE decreases glutamate aspartate transporter (GLAST) expression and/or function, decreasing normal glutamate removal by astrocytes, with consequent increased neuronal activation, augmenting pathological neuroimmune interactions.

While exact mechanisms underlying the long-term impacts of moderate PAE on the CNS and the neuroimmune system are currently under investigation, a summary of several putative mechanisms based on available supporting evidence is discussed below. Additionally, Figure 2 depicts a proposed working model of the possible long-term alterations of different components of the neuroimmune system due to PAE.

### Epigenetics-Steady State Alterations

The term “epigenetic” refers to stable, but potentially reversible alterations of genetic information that result in changes in gene expression, but do not involve changes in the DNA sequence itself (e.g., a lack of genetic mutation). This includes DNA modifications and its regulatory factors such as chromatin structure and actions of non-coding RNAs (ncRNAs) (84). Notably, cell fate specificity and differentiation are often related to epigenetic modification. An epigenetic pattern is closely associated with and responsive to environmental cues. With these properties under consideration, epigenetic modification(s) may meditate negative outcomes as a consequence of adverse *in utero* environmental signals such as alcohol exposure. Epigenetic modification can potentially create long-term reprogramming of gene expression where the initial insult to the fetus is long gone, as observed with FASD.

Emerging clinical evidence of epigenetic modification due to PAE provides a possible mechanism for the enduring effects of PAE (85). For example, an important layer of epigenetic regulation is through ncRNAs such as microRNA (miRNA) and long non-coding RNA (lncRNA). miRNA and lncRNA do not undergo translation, but instead, are involved in various aspects of post-transcriptional modification such as inhibiting mRNA translation to protein, mediating alternative splicing events and promoting post-transcriptional modification of different RNA species. Thus, miRNAs have become an active area of PAE research, specifically in the search for a reliable biomarker for PAE (86). For example, a characteristic miRNA signature was recently identified in plasma collected during pregnancy from moms who gave birth to infants affected by PAE that displayed signs of FASD relative to infants not affected and non-alcohol exposed infants. This study identified increases in eleven miRNAs (e.g., hsa-miR-222 5p, hsa-miR-187-5p, and others) in maternal plasma as potential predictors of worsened outcomes in children with heavy PAE (87).

Few studies exist that link long-lasting alterations in miRNA expression in the brain due to moderate PAE (88, 89). However, while evidence of PAE-related epigenetic modification of immune-related gene expression is sparse, Valles et al., reported that exposure to alcohol (105 ± 45 mg/dl) induced hypermethylation of GFAP (astrocyte structural protein) DNA in fetal brain with a concomitant reduction in GFAP mRNA stability and expression (90). Distinct DNA methylation patterns have also been observed in adolescents and children with FASD (91, 92). Based on the existing and emerging evidence, aberrant DNA methylation, regulation of ncRNA, covalent modification to mRNA, and different ncRNA species may likely play a vital role in the etiology of neuroimmune alterations observed later in life due to PAE.

### Toll-Like Receptor 4 (TLR4)—CNS Actions

Like other peripheral immune cells, innate immune cells in the CNS express receptors that recognize pathogen-associated molecular patterns such as TLRs and NOD-like receptors (inflammasome NLRs). Data from *in vitro* and *in vivo* studies support that alcohol-mediated neuroinflammation involves downstream signaling following TLR and NLR activation (93, 94). Chronic ethanol treatment (87–140 mg/dl) in adult female mice can activate TLR4 signaling on glial cells, thereby inducing the production of proinflammatory molecules and the upregulation of both CD11b and GFAP (93). In addition, microglia from adult alcohol-treated mice affect neuronal apoptosis (e.g., increases) *via* TLR4-dependent pathways (95). Furthermore, TLR-deficient mice given chronic alcohol exposure as adults, exhibit fewer alcohol-mediated cognitive and anxiety-associated behavioral impairments than wild-type mice expressing the TLR4 receptor (96). Moreover, relevant to post-transcriptional regulation, chronic ethanol treatment in adult mice leads to epigenetic modifications in different brain regions in a TLR4-dependent manner (96). Therefore, TLR4-dependent signaling has been linked to alcohol-induced neuroinflammation and associated behavioral and cognitive deficits in an adult-drinking animal model (96). Similarly, in a model of moderate PAE, it has been shown that alcohol-induced microglial activation and neurodevelopmental alterations are mediated by TLR4 signaling (48). To date, a gap in knowledge exists addressing whether *in utero* exposure of *moderate* alcohol alters TLR and NLR expression and/or function specifically on glial/immune cells. While speculative, it is possible that moderate *in utero* alcohol exposure alters TLR functional responses and their co-receptors (e.g., CD14-co-receptor of TLR4), rendering glia and peripheral immune cells highly responsive to further immune activation with consequent augmentation of CNS proinflammatory responses.

### Altered Blood-Brain Barrier (BBB)/Spinal-Barrier Permeability

Another possible mechanism by which moderate PAE may enhance neuroinflammation is via modification of BBB function, resulting in increased permeability. A fundamental component of the BBB is the neurovascular unit, which is made up of neurovascular endothelium, basal lamina, pericytes, and astrocytic end-feet. During inflammation, activated glial cells release factors that further activate each cellular component of the neurovascular unit. The BBB is also responsive to circulating peripheral cytokines and oxidative stress, which alert glial cells residing in the CNS of either pathogen invasion or tissue damage (97). Therefore, integrity of the BBB and the neurovascular unit can shape the course of CNS inflammation (98). As of yet, a lack of clear evidence exists confirming whether moderate PAE alone (i.e., without a later-life insult or challenge) underlies peripheral leukocyte infiltration of the adult CNS. However, one report demonstrated that PAE rat offspring whose mothers’ achieved average serum ethanol concentrations of 140 mg/dl displayed altered morphological development of the glia limitans, a structure consisting of astrocyte end-feet in contact with the pia mater and capillary endothelial cells (99). This study concluded that PAE might induce defects in the glial limitans resulting in leptomeningeal heterotopia. In a separate study, PAE was found to upregulate the vascular cellular adhesion molecule, VCAM-1 in the brain (54). We propose the tentative argument that PAE may cause subtle leakiness at the neurovascular unit leading to chronic low-level glial reactivity. In turn, low-level gliopathy may allow the BBB to reach a threshold of permeability more readily (even with mild immune activation) thereby facilitating greater CNS leukocyte trafficking following subsequent immune challenges (Figure 2). Indeed, the notion of increased leukocyte trafficking was observed following peripheral nerve damage in PAE adults (52). However, it is important to consider that pericytes and astrocytes are heterogeneous in different CNS regions. Moreover, astrocytes are capable of assuming different morphologies and influence differential endothelial cell expression of tight-junction proteins that are critical for barrier formation between endothelial cells. For example, the blood spinal cord barrier is thought to be more permeable than the BBB because of the low number of pericytes and the reduced expression of tight- and gap-junction proteins (100). Therefore, structure and function of the neurovascular unit may be differentially affected by PAE in various CNS regions reflecting differences in PAE-induced cytokine responses (9, 45).

### Peripheral Immune System Dysregulation

A strong and dynamic interplay exists between the CNS and the peripheral immune system (37). Specifically, during disease states, altered BBB permeability can provide improved access for circulating peripheral leukocytes that are able to directly interact with glia and neurons (36, 101, 102). Therefore, PAE-induced alterations of the peripheral immune system can be a strong modulator of CNS-immune interactions.

Clinically, fetal alcohol effects on peripheral immune competence have been suspected for a long time and multiple experimental studies support the possibility that PAE leads to long-term adverse effects on peripheral immune function (23, 103, 104). Dysregulation of cell-mediated immune responses have been reported with diminished T cell-proliferative responses (103, 105) and reduced antigen specificity of T cells (106). PAE has been associated with increased severity in influenza virus infection (106) and autoimmune arthritis (105) and a susceptibility to developing prostate cancer (107).

Collective evidence from other recent studies suggests that the PAE-related alteration of peripheral immune responses goes beyond T cells (52, 53). Published reports from our laboratory indicate that peripheral immune cells, especially myeloid cells (CD11b^+^), seem to be partially activated during young adulthood with moderate PAE, as indicated by increased MHC2 and adhesion molecule expression along with increased cytokine (CCL2) production following *ex vivo* stimulation of splenic leukocytes (52). An extension of this initial work demonstrated that PAE-induced peripheral immune cell activation persists until late adulthood (53). While no significant change was observed in the overall PAE-derived T and B cell numbers compared with controls, basal increases in natural killer cells, and myeloid leukocytes were observed in secondary lymphoid organs. These results indicate possible basal activation of myeloid and natural killer cells in older animals despite the absence of immune challenge. In the same report, peripheral leukocytes (splenocytes and peritoneal leukocytes) from PAE rats displayed exaggerated expression of proinflammatory cytokines such as TNF and IL-1β following *in vitro* immune stimulation (53). Similarly, splenocytes from PAE offspring revealed augmented IL-1β production following *in vivo* LPS treatment (12).

During pathological conditions, a slightly different profile emerges. In the presence of nerve injury, PAE augments peripheral immune cell-derived proinflammatory cytokines IL-1β, TNF, and IL-6 at the site of nerve injury with a concurrent deficiency in IL-10 production (Figure 1A) (52). This PAE-induced exaggerated proinflammatory cytokine production could be due to reduced anti-inflammatory activity from multiple cellular sources and/or dysregulation of required interactions that occur among different immune cell subsets. For example, increased IL-1β and other proinflammatory cytokines produced by innate immune cells can bias increased differentiation of proinflammatory T cells (such as Th17 cells) (108, 109) and decrease immune inhibitory T regulatory (Treg) cells (110). Therefore, while speculative, it is possible that following subsequent inflammation during adulthood, PAE leads to a shift in T cell responses that are biased toward Th17- or Th1-like phenotypes and less toward a Treg phenotype. A possible Th17 bias may underlie a PAE-induced propensity to mount a proinflammatory response. Together, these studies indicate that peripheral immune cells are primed by low to moderate PAE leading to aberrant peripheral immune responses, which may underlie a susceptibility to developing autoimmune disease and inflammatory conditions following immune challenge in adulthood. If true, PAE produces a lifelong vulnerability to develop chronic immunopathological conditions.

### PAE and Glutamate-Mediated Excitotoxicity

Glutamate, one of the best-studied excitatory neurotransmitters, plays a central role in the complex communication networks between neurons and glial cells. Glial glutamate transporters are crucial to ensure glutamate uptake after synaptic release in order to maintain glutamate homeostasis and avoid excessive neuronal excitation. Evidence exists that glutamate aspartate transporter (GLAST), an astrocyte-specific glutamate transporter, is dysregulated under neuroinflammatory conditions (111). Alterations of glutamate reuptake and loss of glutamate transporters are associated with the presence of activated microglia and endangered neurons. Furthermore, proinflammatory mediators such as TNF (produced from glial and peripheral immune cells) can downregulate GLAST leading to impaired glutamate uptake activity (112). Additionally, a number of reports show aberrant glutamate transporter function is strongly linked with neurological disorders. For example, spinal glutamate transporter inhibition results in pathological pain thought to be caused by reduced glutamate clearance and enhanced glutamate-mediated excitation of spinal pain projection neurons (113). Similarly, in amyotrophic lateral sclerosis and multiple sclerosis, dysregulation of glutamate uptake is thought of as a potential mechanism of inflammation in the CNS (112, 114). It is notable that moderate pre- and postnatal alcohol exposure (throughout gestation until weaning at PND22) decreased the expression of GLAST (115). A decrease in glutamate uptake was also observed in hippocampal slices of these adolescent rats (115). Hence, a growing body of evidence indicates that PAE alters glutamate release and clearance by glial cells leading to excessive synaptic and peri-synaptic glutamate (Figure 2). Together, these events may contribute to an excessive neuroinflammatory microenvironment.

## Summary and Conclusion of Current Findings

To summarize, it is evident that PAE poses long-term consequences for neuroimmune function by reprogramming immune activity in the CNS and in the periphery. Therefore, moderate PAE may be a risk factor for various neurological diseases that involve immune-neuroimmune interactions. Though multiple studies report altered neuroimmune responses due to moderate PAE, most of these studies restricted their examination to a microglial role in producing inflammatory cytokines in the CNS. However, endothelial cells and astrocytes also produce and express receptors for many of the same cytokines and have been shown to be important in CNS-immune responses. Currently, a limited amount data exist identifying cell-specific contributions of PAE-related neuroinflammation. A better understanding of immune cell and astrocyte-specific roles may delineate PAE-related mechanisms underlying chronic CNS disease throughout the life span.

While vast majority of initial research in FASD has been conducted to explore the mechanisms of alcohol-induced neurotoxicity due to binge or high exposure of alcohol, in recent years, diverse effects of *moderate* PAE on the immune and neuroimmune systems have drawn significant attention. This area demands more extensive research given the fact that a significant percentage of the human population diagnosed with FASD does not readily display overt early-life indicators of PAE. Yet, these individuals remain vulnerable to impaired CNS function during childhood and likely throughout adulthood. Our current knowledge is based on, a significant amount of research on PAE and neuroimmune interactions driven by previous findings from heavy alcohol exposure (*in utero* and chronic alcoholism in adults). Therefore, extensive research using animal models, as well as careful dissection of existing data from high versus low PAE-induced effects on the developing and adult neuroimmune system will significantly enrich the field. Such work will aid in the identification of key neuroimmune factors underlying FASD, with the possible development of appropriate interventions that could significantly improve the quality of life for these individuals.

## Author Contributions

SN and EM prepared the manuscript and figures, provided substantial contribution to the intellectual content of the manuscript and figures.

## Conflict of Interest Statement

The authors declare that the research was conducted in the absence of any commercial or financial relationships that could be construed as a potential conflict of interest.
